# Data Requirements for Implementing the “Essential-Use” Concept in Chemical Legislation

**DOI:** 10.1021/acs.est.4c10866

**Published:** 2025-06-02

**Authors:** Romain Figuière, Zhanyun Wang, Juliane Glüge, Martin Scheringer, Armin Siegrist, Ian T. Cousins

**Affiliations:** † Department of Environmental Science, 7675 Stockholm University, SE-10691 Stockholm, Sweden; ‡ Empa—Swiss Federal Laboratories for Materials Science and Technology, Technology and Society Laboratory, 9014 St. Gallen, Switzerland; § Institute of Biogeochemistry and Pollutant Dynamics, ETH Zürich, 8092 Zürich, Switzerland; ∥ Institute of Environmental Engineering − Chair of Ecological Systems Design, 27219 ETH Zürich, 8093 Zürich, Switzerland; ⊥ Institute of Food Nutrition and Health − Laboratory of Sustainable Food Processing, ETH Zürich, 8092 Zürich, Switzerland

**Keywords:** REACH Regulation, Stockholm Convention, Sound management of chemicals, REACH Authorisation process, Essential-use concept, Microplastics

## Abstract

The Stockholm Convention and the EU REACH Regulation are two key pieces of legislation on chemicals at the global and European levels, respectively. Discussions have taken place on revising them. For instance, the European Commission is considering implementing the “essential-use” concept in the REACH Regulation to guide decision-making for phasing-out the use of the most harmful chemicals. By assessing 34 existing cases under the Stockholm Convention and 45 restrictions and 544 applications for authorization under the REACH regulation (as of November 2023), this study aims to capture how the essential-use concept may inform decision-making on exemptions and provide insights on its implementation. By conducting a detailed case study of the REACH restriction on intentionally added microplastics, this study also aims to explore how the existing data requirements in regulatory processes could be used in an essentiality assessment. Overall, this study suggests that the Stockholm Convention and the REACH Regulation already consider elements of the concept in their decision-making and that no drastic changes in the data requirements are necessary to apply the concept in decision-making processes.

## Graphical abstract

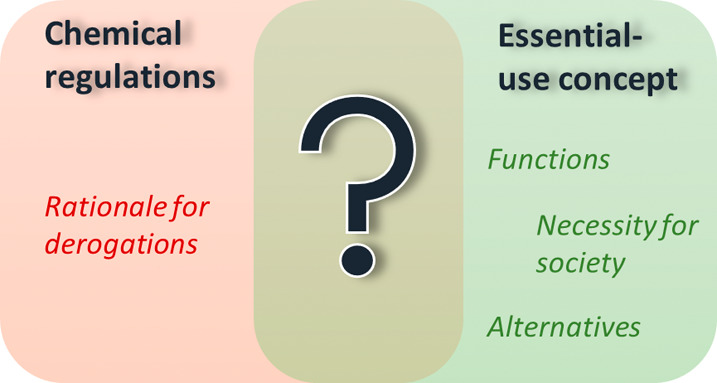

## 1 Introduction

Pollution, climate change, and biodiversity loss have been recognized as a triple planetary crisis to humanity and the environment.^1^ Chemical pollution becomes especially problematic on a global scale when highly persistent substances are emitted. These persistent substances remain intact in the environment for a long period of time and may become globally distributed.^2–4^ Driven by public and scientific concern, regulatory frameworks have been established for the sound management of chemicals at different levels. Two important chemical legislations are the global Stockholm Convention on Persistent Organic Pollutants (POPs) and the European Union (EU)’s REACH Regulation – Registration, Evaluation, Restriction and Authorisation of Chemicals.

Adopted in 2001, the Stockholm Convention is a global treaty to protect human health and the environment from POPs.^5^ In addition to the initial “dirty-dozen” POPs, the Convention includes a five-step process to list new chemicals. The process starts with the nomination of a chemical by a Party which triggers several steps of scientific and socio-economic assessment by the POPs Review Committee (POPRC).^6^ The qualitative socio-economic analysis included in the Convention assesses feasibility, costs, and impacts of control measures. The POPRC then makes recommendations on whether to eliminate or restrict the chemical(s) to the Conference of the Parties (COP), the Convention’s governing body. In its recommendations, POPRC can include specific use(s) and related production of the chemical(s) to be exempted from the Convention’s measures. Then, the COP makes decisions on the listing based on the POPRC recommendations and any additional information presented by Parties and observers. As of November 2023, 34 chemicals or groups of chemicals were listed under the Convention, and 4 more were under evaluation or ready for decisions by COP.

The REACH regulation entered into force in 2007 (Regulation (EC) 1907/2006).^7^ It includes three key components: registration of chemicals; evaluation for their safety; and when risks associated with specific substance(s) are not adequately controlled, their uses may be restricted through the authorization or the restriction processes.^8^ When a substance is identified as a Substance of Very High Concern (SVHCs) and subsequently included in Annex XIV of REACH, it will be subject to the Authorisation process.^9^ As of November 2023, 476 substances were identified as SVHCs,^10^ and 59 entries covering 141 of those substances are in Annex XIV of REACH for authorisation.^11^ In these cases, the European Commission sets a sunset date to cease uses of the substances covered by the Authorisation process. If a company wishes to continue using the substance after the sunset date, it must apply for authorisation, demonstrating either that the risks from its use of the SVHC are adequately controlled (so-called “adequate control route”) or that the socio-economic benefits linked to the use of the SVHC outweigh its risk (so-called “SEA route”) and that no suitable alternative substances or technologies are available to the applicant before the sunset date.^12^

Alternatively, a restriction may be applied, usually limiting or banning the manufacture, placing on the market, or the uses of specific substance(s).^13^ For substance(s) proposed for restriction, a restriction dossier is first prepared by the Competent Authorities (i.e., by the EU Member States or the European Commission), including information on hazards and risks, on availability of alternatives, and a justification for a restriction at the EU level. The dossier submitter must also demonstrate the effectiveness and proportionality of the proposed restriction and whether it is practically enforceable and monitorable. It can also present so-called “derogations” of certain uses to potentially be exempted from the restriction. The restriction dossier is then open for public consultation. Building on the dossier and associated public consultations, the Risk Assessment and Socio-economic Analysis Committees of the European Chemicals Agency (RAC and SEAC) then develop opinions and submit them to the European Commission. In particular, based on an analysis of potential alternatives and a socio-economic analysis, SEAC provides its view on the uses to be potentially exempted. The Commission then takes the final decision on the restriction and exemptions under REACH. As of November 2023, a total of 78 entries of restrictions were added to the Annex XVII of REACH, covering 2127 substances.^14,15^

Recently, discussions have taken place on potential revisions of these existing pieces of legislation on chemicals. For example, the Stockholm Convention does not specify criteria for determining exemptions but only requests specific information as outlined in Annex F for consideration, including: efficacy and efficiency of possible control measures, alternatives, positive and/or negative societal impacts of possible control measures, waste and disposal implications, and status of control and monitoring capacity.^16,17^ It is noted that for several recent listings, the COP agreed to more exemptions than what was recommended by POPRC. In some instances, exemptions go beyond those evaluated by POPRC, while in others, uses rejected by POPRC are granted exemptions by the COP. It has been proposed to delve into the reasons behind these decisions to identify improvements for future practice.^17^ Further, while the processes under EU REACH have led to a decrease in the number of SVHCs on the market,^18,19^ the European Commission recently concluded in the second REACH review that the Authorisation process is too heavy and inflexible and that the restriction process is too slow to sufficiently protect consumers and professional users.^20,21^ Therefore, the Commission wishes to revise both processes to achieve a higher level of protection of human health and the environment.^22,23^ To that end, the Commission considers implementing the “essential-use” concept as a tool for both processes under REACH.^23^

The essential-use concept was included in the Montreal Protocol for phasing out ozone-depleting substances, except for certain essential uses. In the Decision IV/25 adopted in 1992, the Parties agreed that a “controlled substance should qualify as ‘essential’ only if: (1) it is necessary for the health, safety or is critical for the functioning of society (encompassing cultural and intellectual aspects); and (2) there are no available technically and economically feasible alternatives or substitutes that are acceptable from the standpoint of environment and health”.^24,25^ In 2019, Cousins et al. suggested further using the essential-use concept to guide the phase-out of per- and polyfluoroalkyl substances (PFASs).^26,27^ Work has been ongoing to seek for clarity on whether and how to operationalize the essential-use concept in the EU chemical regulations to guide decision-making to phaseout the use of the most harmful chemicals.^28–31^ In the latest guidance published by the European Commission, three main questions were proposed to be considered for evaluating the essentiality of a use: (1) Is the technical function of a most harmful substance needed for the final product to deliver its service? (2) Does the use of this most harmful substance fulfill at least one of the criteria listed in the guidance to be considered as necessary for health and safety or critical for the functioning of society? And, (3) are safer alternatives capable of providing similar function and sufficient level of performance available?^28^

In this study, we explore two key aspects related to a potential implementation of the essential-use concept in legislation: how its application might inform decision-making on exemptions, and how existing data requirements in regulatory processes could be used within an essentiality assessment. To address these aspects, we first analyze existing cases under REACH and the Stockholm Convention (as of November 2023), with a focus on justifications for individual derogations, authorizations, or exemptions, as well as any negative cases. We then conduct a detailed case study, using the REACH restriction dossier on intentionally added microplastics, to assess the applicability of existing data requirements in regulatory processes for an essentiality assessment. The findings of our analysis aim to support the ongoing development of the essential-use concept for operationalization in legislation.

## 2 Methods

### 2.1 Evaluation of Existing Regulatory Outcomes

The READ approach, a four-step method specifically developed for systematic analysis of policy documents,^32^ was followed to determine how existing regulatory outcomes under REACH and the Stockholm Convention match the essential-use concept and to identify reasons for discrepancies.

*Step 1—Readying the materials.* In this first step, relevant policy documents were selected for analysis, covering both recommendations made by the relevant scientific subsidiary bodies and final regulatory decisions taken by the policy-making bodies.

Under the Stockholm Convention, all final risk management evaluations prepared by POPRC and the corresponding final decision adopted by the COP^33^ were selected. Similarly, for the REACH restrictions, the final SEAC opinions on all previous restriction proposals^14^ and their associated final legal decision adopted by the European Commission were selected.^14^

For the REACH authorizations, in addition to the final SEAC opinions and related European Commission decisions of all previous authorization cases, a list of the short-listed alternatives evaluated by companies in their applications for authorization, published by the European Chemicals Agency (ECHA), was taken into account. This list summarized the reasons for rejections of the alternatives according to the applicants, and whether they identified “most promising alternatives” for the use they applied for as of June 2023.^34^

*Step 2—Extracting the data.* Information on the derogations/exemptions that were recommended or rejected by SEAC or POPRC, and for which reasons, was manually collected, as well as the lists of derogations/exemptions in the final legal decisions. Regarding the Authorisation process, information was collected to determine the argumentation route that was used by the applicant as justifications, i.e., the “SEA route” or the “adequate control route”. All the data collected are available in the Supporting Information (Tables S1.1, S1.2, and S1.3).

*Step 3—Analyzing the data.* The extracted data were manually analyzed to capture the rationale for recommending or rejecting a derogation/exemption/authorization by SEAC or POPRC. Then, the rationales were analyzed by grouping them into the following four categories to evaluate how they could be related to elements of the essential-use concept: (1) referring to the necessity of the technical function for the performance of the end-product; (2) referring to the necessity of the use for health and safety or its criticality for the functioning of society (according to the criteria recommended by the European Commission, Tables S2.1 and S2.2); (3) referring to the lack of suitable alternatives; and (4) referring to other reasons (e.g., negligible releases/exposure/risks, the chemical(s) present as impurities in other products, costs outweigh the benefits). All the key information, including the exact quotes from the SEAC final opinions and the POPRC Risk Management Evaluations referring to the reasoning for recommending derogations (or not) (Tables S1.1 and S1.2) and related to the applications for authorization (Table S1.3), were stored in the Microsoft Excel spreadsheets provided as the Supporting Information.

*Step 4—Distilling the findings.* The analysis was further interpreted using basic tools (e.g., pivot tables) to evaluate how the current regulatory outcomes match or differ from the essential-use concept. It is important to note that at this stage, the objective is 2-fold: to determine (1) how an application of the essential-use concept may inform decision-making under existing regulatory frameworks, and (2) whether the information used by POPRC and SEAC to justify an exemption could be used in an essentiality assessment. It is not to re-evaluate whether existing exemptions would be “essential” according to the latest guidance from the European Commission (such a re-evaluation was conducted only in the case study below).

### 2.2 Case Study

The restriction on the intentional use of microplastics under REACH served as a case study for further in-depth analysis. The essential-use concept was applied, following the authors’ interpretations of the approach recently released by the European Commission,^28^ to the same information in the relevant public documents that were available to SEAC and POPRC, using the following three main steps:

*Step 1 - Assessing the necessity of the technical function of microplastics for the final product to deliver its service*. The functional-substitution concept was followed to determine whether microplastics are necessary for the technical performance of the final product^35,36^ (the term “product” is used in its broad sense, including processes, preparations, and articles in a typical regulatory context). To do so, the information available in the restriction dossier was analyzed to determine the product types and the uses of the targeted polymers, including the technical function they provide, their associated end-use function, and their associated function as a service, as defined in a previous study.^36^ Definitions of the terms are available in Table 1.

**Table 1:** Definitions for Specific Terms

**Term**	**Definition**
Use	General description of the sector and types of products in which the microplastics are used in (e.g. printing)
Specific application	Specific examples of products and processes (e.g. printing on thermal paper)
Technical function	Determined by its physicochemical properties, it describes what the chemical is used for (e.g., bisphenol A as a chemical developer)^36^
End-use function	Function provided by the substance in the context of a specific product/industrial process (e.g., the use of bisphenol A in thermal paper for the creation of a printed image)^36^
Function as a service	Service provided by the product (e.g., record of a sale by printing cash register receipts using thermal paper)^36^

Step 2 - *Assessing the necessity of the function of the target chemicals for health and safety and for the functioning of society*. The list of possible criteria recommended by the European Commission, listed in the Supporting Information (Tables S2.1 and S2.2), was used for the assessment.^28^

*Step 3 - Assessing the availability of safer alternatives.* It was assumed that alternatives were available for those uses for which SEAC recommended a derogation due to reasons other than a lack of suitable alternatives. Additionally, the information available in the restriction dossier was analyzed to determine whether the safety of potential alternatives had been evaluated.

## 3 Results and Discussion

The following subsections present an overview of the regulatory outcomes under the Stockholm Convention, as well as the REACH Restriction and Authorisation processes. This is followed by an analysis of the main arguments used by POPRC and SEAC to justify the need for exemptions and how these arguments are related to the “essential-use” concept. Subsequently, we present the findings of our analysis on the essentiality of uses of intentionally added microplastics, based on our interpretation of the European Commission’s approach.

### 3.1 Overview of the Regulatory Outcomes Analyzed, as of November 2023

Under the Stockholm Convention, 22 substances or groups of substances had been listed additionally to the “dirty dozen” by the time of this analysis. For 9 of them, no exemption was recommended by POPRC to the COP. In contrast, POPRC recommended 48 use exemptions for the rest of the listed substances. Perfluorooctanesulfonic acid (PFOS), its salts and perfluorooctanesulfonyl fluoride (hereafter referred to as “PFOS” only), perfluorooctanoic acid (PFOA), its salts and PFOA-related compounds (hereafter referred to as “PFOA” only), and short-chain chlorinated paraffins were the three listings with the highest number of use exemptions that were evaluated by POPRC (i.e., 13, 11, and 9, respectively).

Under REACH, the SEAC has issued final opinions on 45 restriction dossiers. For 14 of them, no derogations were proposed by the dossier submitter, which was kept by SEAC. For another two, derogations were proposed by the dossier submitter, but SEAC did not recommend them in its final opinions. In contrast, the SEAC has recommended derogations for 184 specific uses in 29 restriction cases to the European Commission. Restrictions on lead in projectiles and in fish sinkers and lures for outdoor activities, PFOA, its salts, and PFOA-related substances, and intentionally added microplastics are the three cases with the highest number of derogations recommended by SEAC (i.e., 24, 22, and 20, respectively).

Furthermore, 544 applications were submitted to ECHA for the authorization of the uses of 34 SVHCs. SEAC has issued opinions on 448 of them, and the European Commission has published a decision on 328 of them. Chromium trioxide and 4-(1,1,3,3-tetramethylbutyl)­phenol, ethoxylated are the two substances for which the highest number of applications for authorization were submitted and evaluated by SEAC (i.e., 145 and 100, respectively).

### 3.2 Reasoning for Granting Derogations/Authorizations/Exemptions in the Existing Outcomes

Generally, POPRC and SEAC listed several arguments, respectively, to justify the need for a derogation. All the arguments including those related to the essential-use concept presented by POPRC and SEAC in their justifications to grant a derogation/exemption are presented in the Supporting Information, categorized by their types (Tables S1.1 and S1.2, respectively).

#### Under the Stockholm Convention

In 28 of the 48 different recommendations of use exemptions, the lack of alternatives to the substance of concern is referred to in the justification. The necessity of the use for health and safety is referred to in 6 recommendations of use exemptions, while the criticality of the use for the functioning of society is never referred to. The necessity of the technical function for the final product is referred to in only one exemption for the use of PFOS in chemically driven oil production.

Linking this reasoning back to the aforementioned three main questions/components in the guidance on the essential-use concept by the European Commission and removing double counting give that one component is referred to in the POPRC justification for 25 of the 48 recommendations of use exemptions (Figure 1A). In another five cases, both the necessity for health and safety and the lack of alternatives are referred to in the reasoning: the uses of PFOA in medical devices (both implementable devices and others) and technical textiles, the use of HBCDD in expanded and extruded polystyrene in building materials, and the use of pentachlorophenol in wood preservatives for utility poles and cross-arms (SI 1.1). Finally, all three components are never jointly referred to in the reasoning of the POPRC to justify an exemption recommendation.

**Figure 1:**
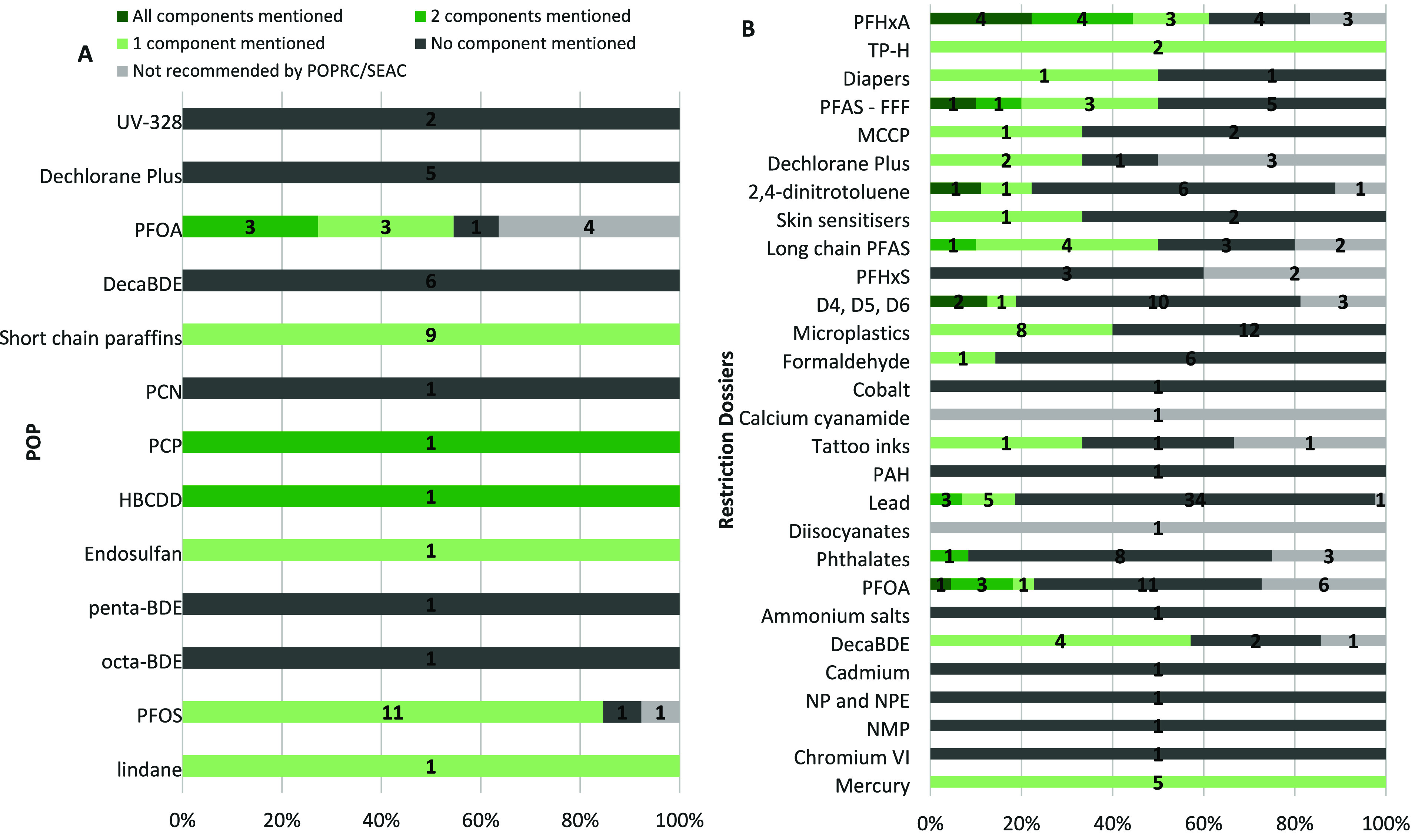
Number of components of the essential-use concept mentioned in the reasoning to grant derogations for uses of substances listed under the Stockholm Convention (A) and in former REACH restriction dossiers (B). *Note: In the legend, “Not recommended by POPRC/SEAC” refers to the number of derogations which were evaluated by POPRC/SEAC but for which they concluded that they should not be granted. On the x-axis: the percentages indicated on the x-axis of the figure represent the share of derogations being discussed for each substance listed or restriction dossier. On the y-axis: to facilitate the reading of the figures, numbers of several restriction dossiers were aggregated as follows: “Lead” represents an aggregation of the data related to 5 different restriction dossiers (i.e., lead in projectiles (for firearms and air guns) and in fishing sinkers and lures for outdoor activities; lead and its compounds in shots for shooting in wetlands; lead and its compounds as PVC stabilizer; lead and its compounds in consumer articles; and lead and its compounds in jewelry).* (A) PFOS: perfluorooctanesulfonic acid; octa-BDE: commercial octabromodiphenyl ether (its components: hexabromodiphenyl and heptabromodiphenyl); penta-BDE: commercial pentabromodiphenyl ether (its components tetra- and hexabromodiphenyl ether); HBCDD: hexabromocyclododecane; PCP: pentachlorophenol and its salts and esters; PCN: chlorinated naphthalenes; (B) NMP: 1-methyl-2-pyrrolidone; NP and NPE: 4-nonylphenol, branched and linear, and 4-nonylphenol, branched and linear, ethoxylated; DecaBDE: Bis­(pentabromophenyl) ether; Ammonium salts: inorganic ammonium salts; PFOA: perfluorooctanoic acid, its salts and PFOA-related substances; Phthalates: diisobutyl phthalate, dibutyl phthalate, benzyl butyl phthalate, and bis­(2-ethylhexyl) phthalate; PAH: Polycyclic-aromatic hydrocarbons; Tattoo inks: substances with a harmonized classification as carcinogenic, mutagenic or reprotoxic (CMR) categories 1A and 1B, or as skin sensitizer used in tattoo inks and permanent makeup; Cobalt: cobalt carbonate, cobalt di­(acetate), cobalt dichloride, cobalt dinitrate, and cobalt sulfate; Formaldehyde: formaldehyde and formaldehyde releasers; Microplastics: intentionally added microplastics; D4, D5, D6: octamethylcyclotetrasiloxane, decamethylcyclopentasiloxane, and dodecamethylcyclohexasiloxane; PFHxS: perfluorohexane-1-sulfonic acid, its salts and related substances; Long chain PFAS: perfluorononan-1-oic acid (PFNA), nonadecafluorodecanoic acid (PFDA), henicosafluoroundecanoic acid (PFUnDA), tricosafluorododecanoic acid (PFDoDA), pentacosafluorotridecanoic acid (PFTrDA), heptacosafluorotetradecanoic acid (PFTDA), including their salts and precursors; Skin Sensitizer: Skin sensitizing, irritative and/or corrosive substances; Dechlorane Plus: 1,6,7,8,9,14,15,16,17,17,18,18-Dodecachloropentacyclo­[12.2.1.16,9.02,13.05,10]­octadeca-7,15-diene; MCCP: medium-chain chlorinated paraffins; PFAS – FFF: per- and polyfluoroalkyl substances in firefighting foams; Diapers: Polycyclic aromatic hydrocarbons (PAHs), polychlorinated dibenzo-p-dioxins (PCDDs), polychlorinated dibenzofurans (PCDFs), polychlorobiphenyls (PCBs), and formaldehyde used in single-use baby diapers; TP-H: terphenyl, hydrogenated; PFHxA: undecafluorohexanoic acid, its salts and related substances.

Furthermore, POPRC used other arguments which are not related to the essential-use concept to justify the needs for 18 use exemptions, including: (1) allowing the possibility to continue using articles containing the substances of concern until their end of life for 10 uses, (2) more time needed for the market to fully transition toward the identified alternatives for 5 uses, and (3) difficulty in implementing the ban for 3 uses. Also, POPRC recommended 5 exemptions without providing details in the corresponding risk management evaluation.

Overall, two observations can be made here. First, in 13 out of the 48 recommendations of use exemptions, POPRC based the recommendations solely on technical reasons related to the enforcement of the Convention, and thus, it is likely that implementing the essential-use concept would not influence such recommendations (Figure 2A). There are, on the other hand, 35 cases where the essential-use concept may have the potential to influence or change the assessment outcomes by POPRC. Second, while POPRC has not recommended any exemptions by assessing the three components of the essential-use concept all at once, individual components of the concept were already separately assessed. This suggests that POPRC has the capacity, in many if not all cases, to obtain relevant information and assess the three components as a whole. Thus, the results suggest the essential-use concept could be readily integrated in the Stockholm Convention to guide POPRC’s assessment of whether an exemption is needed without significantly modifying the current process.

**Figure 2:**
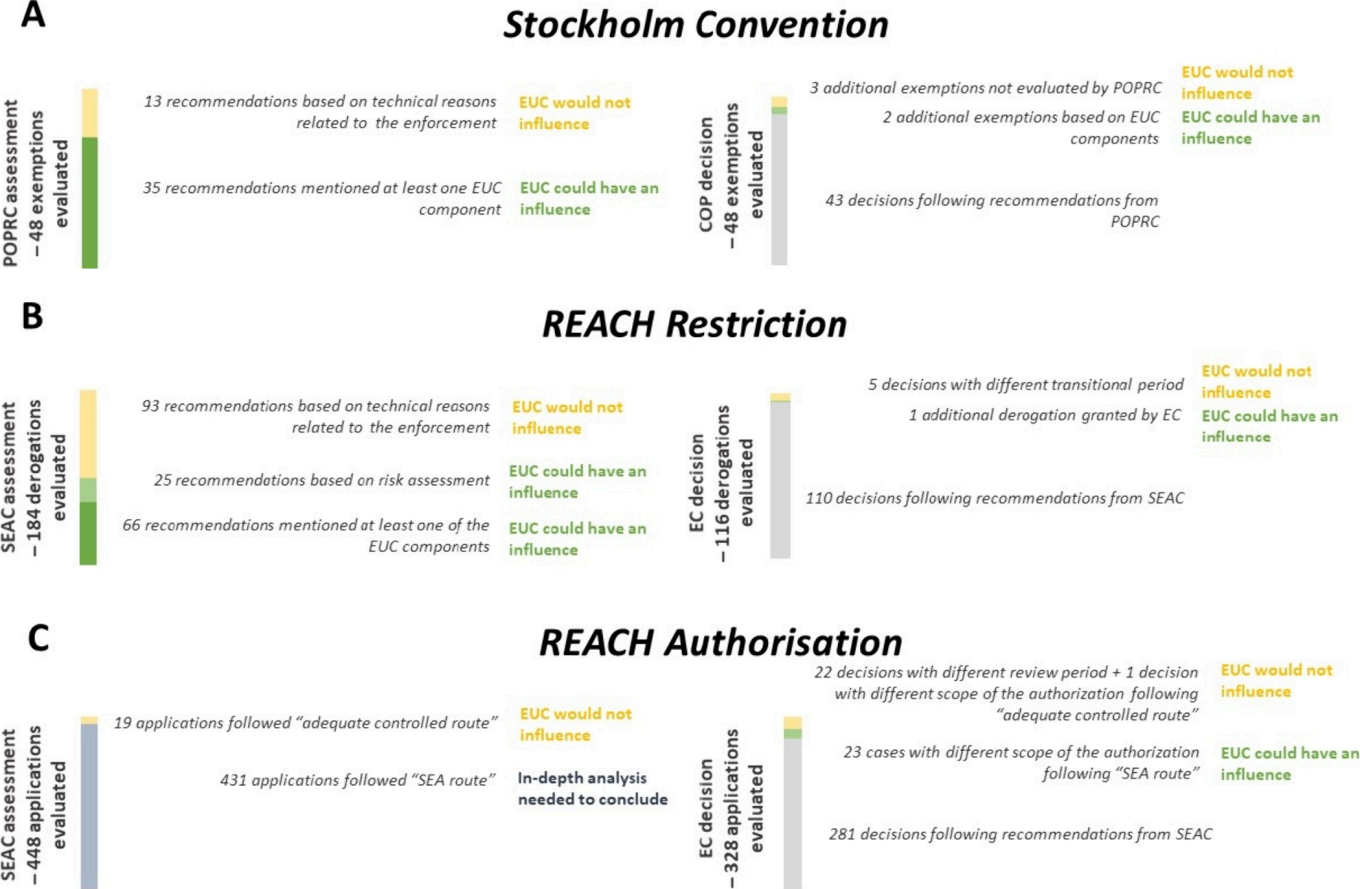
Overview of the number of derogations for which the essential-use concept could have influenced the decision-making under (A) the Stockholm Convention, (B) the REACH Restriction process, and (C) the REACH Authorisation process.

Further, by taking a closer look at the final COP decisions vis-a-vis the POPRC recommendations, some discrepancies can be observed (Figure 2A). For example, for the uses of PFOA in membranes intended for medical textiles and filtration in water treatments, and in photoimaging applications, the COP granted time-limited exemptions, while POPRC recommended no exemptions as the phase-out of PFOA in these uses has been observed. This is because some parties insisted on the lack of alternatives for their domestic manufacturers, which raises the question of how the availability of alternatives should be considered in the essential-use concept (e.g., when handling substances of concern, whether it is necessary to delay the phase-out until all individual companies have found alternatives).

Furthermore, the COP additionally granted exemptions for three use cases, although these uses were not assessed by POPRC. In such cases, implementing the essential-use concept alone to guide recommendations of POPRC would not change such decision-making, but other adjustments of the process would also be needed to ensure all relevant uses are assessed by POPRC.^17^

#### REACH Restriction Process

For the 29 restriction dossiers for which SEAC recommended derogations, the lack of suitable alternatives is referred to in the reasoning to justify 57 of the 184 derogations recommended by SEAC, followed by 22 recommendations referring to the necessity of the use for health and safety and 6 recommendations referring to the criticality of the use for the functioning of society. The necessity of the technical function delivered by the substance(s) of concern for the final product was referred to in the SEAC reasoning for 14 recommendations.

Taking a closer look at these reasoning in relation to the essential-use concept and removing double-counting, at least one component of the concept is referred to by SEAC in the reasoning for 66 derogations recommended (Figure 1B). For 9 derogations, all three components are referred to in the SEAC reasonings: the use of PFOA in nonimplementable medical devices, use of D5 and D6 in medical devices, use of D5 for professional use in the cleaning and restoration of art and antiques, use of 2,4-dinitrotoluene for micro gas generators in seat belt pretensioners, uses of PFHxA in medical devices, personal protective equipment, high performance separation media, and fluoropolymers, and use of PFASs in firefighting foams for establishments covered by the Directive 2012/18/EU. Our analysis suggests that SEAC may have the capacity to obtain relevant information and assess whether a use is essential, provided that clear and robust guidance is available on how to appropriately assess such information. From a data requirements perspective, this indicates that the essential-use concept could be feasibly integrated into the current Restriction process to support decision-making. These findings are in line with a previous study which suggested that an essentiality assessment could be based on socio-economic benefit analysis within REACH.^29^ At the same time, further work is needed to investigate how the concept should be implemented in practice, including an assessment of whether decisions following the essential-use concept would align with or potentially conflict with other European legal principles.

Furthermore, SEAC has used other reasons than the components of the essential-use concept to justify 160 derogations, including 8 main subcategories of arguments: (1) the particular use is already regulated elsewhere (20 cases); (2) the enforcement of the restriction on the use would be too difficult (16 cases); (3) the particular use is outside of the scope of the restriction (22 cases); (4) the substance of concern is present as impurities in the particular use (18 cases); (5) more time is needed for the market to transition toward an alternative solution (30 cases); (6) more time is needed to allow the continued use of articles until their end of life (14 cases); (7) the estimated releases, exposure, and/or risk related to the use are expected to be negligible (24 cases); and (8) the costs of the phase out of the substance of concern for the particular use outweigh the risk (7 cases). For the remaining 9 recommendations of derogations, the reasoning was tailored to the individual specific uses and could not be generalized.

Among these 160 derogation recommendations, 118 did not refer to any components of the essential-use concept. Most (93) of them are agreed upon the basis of technical reasons specific to enforcement (e.g., the use is already regulated elsewhere; the use is outside of the scope of the restriction; more time is needed to allow the continued use of articles until their end of life). It is likely that such justifications related to the enforcement of chemical regulations would still be relevant to justify granting a derogation, even if the essential-use concept is implemented. In other words, implementing the concept would likely not change such recommendations (Figure 2B).

Meanwhile, 25 derogations are recommended based on the following: (1) it was believed the resulting risk was negligible, or (2) the costs of applying the restriction would outweigh the risk of the continued use. However, as demonstrated in previous studies, a risk-based approach is not appropriate to manage chemicals with long-term effects, like PBT and vPvB substances.^37,38^ In such cases, implementing the essential-use concept could imply that derogations for uses of the most harmful substances would no longer be based on a risk assessment but based on the essentiality of specific use(s). Therefore, implementing the essential-use concept could influence the decision-making by removing nonessential uses of such substances (e.g., up to 13.8% of the cases analyzed).

The European Commission followed the SEAC recommendations in 110 cases, whereas in six cases, discrepancies are present in the following three types: (1) two cases with a shorter transitional period than in the SEAC opinions; (2) three cases with a longer transitional period, and (3) one case with derogations for which SEAC concluded no need for derogations. In the former two types of cases, implementing the essential-use concept would likely not change the decision-making, whereas in the last type of case, implementing the concept could make a difference (Figure 2B).

#### REACH Authorisation Process

Under REACH, an authorization may be granted if the applicant successfully demonstrates that (1) the risk from the use of the SVHC is adequately controlled (so-called “adequate control route”); or (2) the socio-economic benefits linked to the use of the SVHC outweigh its risk (so-called “SEA route”), and that no suitable and economically feasible alternative substances or technologies are available to the applicant before the sunset date.^12,39^ Therefore, every decision to grant an authorization is justified by a lack of suitable alternatives plus another reason which is not necessarily related to the components of the essential-use concept. In 79 cases, the applicants stated that they had identified a “most promising alternative” in their application for authorization; in these cases, we concluded that SEAC has recommended to grant an authorization because more time is needed for the transition. Implementing the essential-use concept would likely not change such recommendations.

A previous study demonstrated that the essential-use concept could be implemented to guide decision-making on the applications for authorization only in those cases where the applicants followed the “SEA route”.^39^ This is because it is illegal for the European Commission to refuse to grant an authorization if the applicant successfully demonstrates that the risk resulting from their use is adequately controlled (i.e., the “adequate control route”), even when the use would be deemed nonessential.^39^ Thus, the essential-use concept could not be applied in the decision-making on the applications for authorization that follow the “adequate control route”; but such cases represent only a minority of the uses that have been applied for thus far (19 out of 448) (Figure 2C).

For the applications that follow the “SEA route”, Figuière et al. (2023) demonstrated that ECHA encourages the applicants to identify the function of the substances of concern and to explain “if and how the final product would be affected by a change in substance/process and the use of alternatives” in their application. They are also encouraged to evaluate the impacts on the consumers, human health, and the environment of a potential loss of function and service provided by the substance of concern in case the authorization would not be granted.^39^ Thus, applicants are already expected to provide adequate information to SEAC to evaluate the essentiality of the use for which they apply for. However, as the quality of the information and the level of detail provided by the applicants are not explicitly defined,^39^ it is difficult to assess whether the essential-use concept would have influenced the decision-making on the authorizations which followed the “SEA route” without an in-depth analysis of existing applications for authorization. Such an in-depth analysis goes beyond the scope of this study.

In the majority of the cases (281 out of 331), the European Commission followed SEAC’s recommendations, while in some other cases, discrepancies are present. In 18 cases (12 uses of lead chromates, one use of trichloroethylene, one use of sodium dichromate, and four uses of chromium trioxide), the European Commission granted an authorization with a shorter review period than the one SEAC recommended on the basis that it was difficult to assume the nonsuitability of the alternatives for a long period and they assumed less time needed by the companies to implement alternatives. For two uses of bis­(2-ethylhexyl) phthalate, the European Commission granted a shorter review period due to some deficiencies in the exposure assessment in the workplace made by the applicants. For two uses of “4-(1,1,3,3-tetramethylbutyl)­phenol, ethoxylated”, the European Commission granted a longer review period than the one SEAC recommended because they disagreed with SEAC’s justifications in setting the length of the review period. Implementing the essential-use concept would not affect such discrepancies.

For 20 uses of chromates, the European Commission granted only a “partial authorisation” as they argued that the SVHCs should not be used for parts of the use applied for where “the key functionality [of the SVHC] is not necessary for the use”. Similarly, the European Commission granted only partial authorization for four additional uses of chromates because the scope of the uses that were applied for was too broad. In such cases, implementing the essential-use concept could avoid discrepancies by more detailed assessments of the uses. The European Commission also granted one partial authorization for the use of bis­(2-methoxyethyl) ether for only the uses of the substance for which the exposure scenarios demonstrated that the risk was adequately controlled. As this particular application followed the “adequate control route”, implementing the essential-use concept could not have avoided such discrepancy for legal reasons as stated above (Figure 2C).

#### Summary

As the components of the essential-use concept have already been assessed in the existing decision-making processes as demonstrated above, decision-making under existing regulations such as EU REACH and the Stockholm Convention could already consider the concept without drastic changes in the data requirements. Implementing the concept can help to further narrow down the range of derogations/authorizations/exemptions by removing some nonessential uses, as already suggested by another study.^30^ In particular, the concept has the potential to inform the decision-making on substances of concern for which it is not possible to reliably determine the risk they pose to the environment and human health by providing a framework centered on the actual purpose of a substance of concern. Most important is that the necessary information on the exact function delivered by the substance of concern must be available to the Competent Authorities in order to apply the essential-use concept. The specific case of the REACH restriction on intentionally added microplastics was used as a case study to determine whether the information provided to the authorities in the restriction dossier was enough for them to evaluate the essentiality of the uses of microplastics.

### 3.3 Case Study on Intentionally Added Microplastics

In the following, we re-evaluate the REACH restriction dossier on intentionally added microplastics,^40,41^ as a case study, to understand whether the information available to the Competent Authorities at the time was adequate for applying the essential-use concept in the decision-making. Background information on the restriction dossier on intentionally added microplastics is provided in the Supporting Information (S3 and S4).

Overall, 32 derogations were proposed by the dossier submitters. Three derogations were proposed for the uses of microplastics in products which are covered by other regulations (i.e., fertilizing products covered by the Regulation (EC) No. 2019/1009, medicinal products, and substances containing food additives).^40,42–44^ Those derogations were recommended for technical reasons specific to the scope of EU REACH, and it is likely that they would still be proposed if the essential-use concept is implemented. Similarly, two derogations were proposed for the uses where microplastics are present as impurities (i.e., in sludge compost, and food and feed products).^40^ Due to its technical nature related to the scope of the restriction being intentionally added, it is likely that these derogations would also be proposed if the concept is implemented.

Furthermore, three derogations were proposed for polymers, which are believed to not represent the same concern as microplastics to the environment.^40^ As these derogations refer to the scope of the restrictions, i.e., the definition of substances of concern), implementing the concept would not affect such derogations. Similarly, a derogation was proposed for substances and mixtures containing microplastics used at industrial sites, as industrial uses were outside of the scope of the restriction. The concept would not affect such derogation either if the dossier submitters decided to focus the restriction on consumer uses when preparing the dossier.

This leaves 23 uses across 10 different product types that were evaluated by the dossier submitter.^40,41^ Our assessment and conclusions of the essentiality of these uses are presented in a separate Microsoft Excel Spreadsheet (Table S5) and summarized in Table 2. For 18 of these 23 uses, adequate information is available in the restriction dossier to determine the technical function of microplastics, their associated end-use function, and their associated service. In the case where microplastics are used in rinse-off cosmetic products which do not contain microbeads, the dossier submitter stated that microplastics are mainly used as opacifiers to give the product a “milky texture” to improve the consumer perception. Thus, we determine that this particular use of microplastics is not necessary for the technical performance of the final product and that it can be directly assigned as nonessential. Similarly, microplastics are used in detergents and maintenance products as fragrance encapsulates to ensure a long-lasting scent after washing. Although a good perfume is nice to have after washing, we assume that it is not necessary for the technical performance of the detergents and maintenance products, which is to clean a surface. Thus, we determine that microplastics used as fragrance encapsulates in those products are not essential. In all other uses (n = 15), the uses of microplastics could be considered as necessary for the technical performance of the final product. It is important to note that in some cases, microplastics provide several functions to the products, which are not all necessary for the final product. For example, nonmicrobead microplastics in detergents and maintenance products are present in the products for their functions not only as rheology modifiers, antifoaming agents, and/or complexing agents but also as opacifiers. Although the former functions could be considered as necessary for the technical performance of detergent products as these functions improve their efficacy, the latter opacifier function only aims at improving consumer perception, which is not considered as necessary.

**Table 2:** Potential Changes on the Decision-Making of Derogations in the Microplastics Restriction Dossier if the Essential-Use Concept Would Have Been Applied

**Derogation as proposed by the dossier** **submitter** ^40^	**Main reason for derogation according to dossier** **submitter** ^40^	**Rational of the authors following the essential-use concept**
***Derogations not evaluated by the author following the essential-use concept***		
**Substances, mixtures, or articles where microplastic is contained by technical means to prevent releases**	Negligible risk/exposure	Assessment of derogations focused on the specific uses, not the potential reduction of emissions
**Substances, mixtures, or articles where the physical properties of the microplastic are permanently modified**	Negligible risk/exposure	Assessment of derogations focused on the specific uses, not the potential reduction of emissions
**Substances, mixtures, or articles where the microplastics are permanently incorporated in a solid matrix**	Negligible risk/exposure	Assessment of derogations focused on the specific uses, not the potential reduction of emissions
***Derogations proposed by the authors related to the scope of the restriction***		
**Natural polymers not chemically modified**	Negligible risk/exposure	Do not meet the definition of the substance of concern
**Polymers which are (bio)degradable**	Negligible risk/exposure	Do not meet the definition of the substance of concern
**Polymers with solubility > 2g/L**	Negligible risk/exposure	Do not meet the definition of the substance of concern
**Substances or mixtures containing microplastics used at industrial sites**	Out of scope of the intended restriction	Out of scope of the intended restriction
***Derogations proposed by the authors because the use is regulated elsewhere***		
**Substances or mixtures regulated under Regulation (EC) No. 2019/1009** **on Fertilising Products**	Already regulated elsewhere	Already regulated elsewhere
**Medicinal products for human or veterinary use**	Already regulated elsewhere	Already regulated elsewhere
**Substances or mixtures containing food additives**	Already regulated elsewhere	Already regulated elsewhere
***Derogations proposed by the authors because microplastics are present as impurities***		
**Sludge and compost**	Microplastic present as impurities	Microplastic present as impurities
**Food and feed**	Microplastic present as impurities	Microplastic present as impurities
***Derogations proposed by the authors because the use is considered as essential***		
***In vitro*** **diagnostic (IVD) devices – Human health applications**	Costs outweigh the risk	Essential use
***In vitro*** **diagnostic (IVD) devices – Veterinary applications**	Costs outweigh the risk	Essential use
**Detergents and maintenance products without microbeads**	Lack of alternatives	Essential use
**Agricultural** **& horticultural uses: Controlled release fertilizers**	Lack of alternatives	Essential use
**Agricultural** **& horticultural uses: Fertilizer additives**	Lack of alternatives	Essential use
**Agricultural** **& horticultural uses: Capsule suspensions in plant protection products**	Lack of alternatives	Essential use
**Agricultural** **& horticultural uses: coated seeds**	Lack of alternatives	Essential use
***Derogations not proposed by the authors because the use is considered as nonessential***		
**Rinse-off cosmetic products containing microbeads**	Time needed for transition	Not necessary for health and safety, nor is critical for the functioning of society
**Other rinse-off cosmetic products**	Time needed for transition	Use of microplastic not necessary for the technical performance of the final product
**Fragrance encapsulates**	Lack of alternatives	Use of microplastic not necessary for the technical performance of the final product
**Leave-on cosmetic products**	Lack of alternatives	Not necessary for health and safety, nor is critical for the functioning of society
***Derogations for which the authors could not conclude on essentiality based on the information available***		
**Medical devices (where microplastics cannot be contained during end** **use)**	Lack of alternatives	Difficult to assess the necessity of the microplastics in the final products

For 15 of the 18 cases where the use of microplastics is necessary for the technical performance of the final product, we have further assessed whether the function(s) provided by microplastics is necessary for health and safety and is critical for the functioning of society. We determine that the uses of microplastic in cosmetic products and in food additives are not necessary for society, as none of these uses meet any of the criteria listed in the communication from the European Commission^28^ and in the Supporting Information (S2). Based on the information contained in the restriction dossier, it is not possible to determine whether the use of microplastics in polish and in paints and coatings is necessary for society as no information about the uses of the final products is provided. For example, paint with friction resistance and antislip effects could be considered as critical for the functioning of society if it is used for road marking, but not if it is used for decorative purposes.

Out of the 10 uses where microplastics are necessary for the technical performance of the final product, necessary for health and safety, and/or critical for the functioning of society, no safer alternatives were available in seven cases; i.e., for microplastics used in agricultural and horticultural products, *in vitro* diagnostic devices, and detergents and maintenance products (if they are not used as microbeads). It is important to note that the dossier submitter did not evaluate whether the potential alternatives to microplastics identified were safer. At best, only a brief qualitative description of the hazard profile of the alternatives is provided in the restriction dossier. For the four uses of microplastics in medical devices, information on the function provided by microplastics in the restriction dossier is insufficient for concluding on the essentiality.

Based on the present analysis, it is likely that the uses of microplastics in cosmetics (both rinse-off and leave-on cosmetic products) and as fragrance encapsulates would not be considered essential and would require no derogations (Table 2). Furthermore, as explained previously, the derogations for uses for which the dossier submitters and RAC believed that releases of microplastics would be negligible would likely not be proposed under the essential-use concept, as the assessment would focus on the use and function of the substance and not on the exposure resulting from its use.

#### Lessons Learned from the Case Study: Opportunities and Challenges of the Essential-Use Concept

Overall, the case study demonstrates that the evaluation of potential derogations following the essential-use concept (in addition to other criteria or as sole criteria) would lead to an assessment that focuses on the functions provided by the substance of concern and its actual benefits to society, rather than on the relevance of the risk a specific use may represent. We could reach a conclusion on the essentiality of 19 out of the 23 uses discussed in the restriction dossier based on the information provided by the dossier submitters, suggesting that enough information is provided in restriction dossiers to perform an essentiality assessment and confirming that no drastic changes in the data and information requirements of the REACH Restriction process are needed to apply the concept in decision-making. Furthermore, we concluded that 17 derogations out of the 24 that were recommended by SEAC would still be recommended by following the essential-use concept. These results suggest that applying the essential-use concept would not drastically change the regulatory outcomes in this particular case.

For seven uses, microplastics provide more than one technical function in the products. In those cases, it might be more difficult to find suitable alternatives, as it is unlikely that one alternative can provide the same combination of functions. However, not all functions provided by microplastics should necessarily be considered necessary for the technical performance of the end product. For instance, microplastics are used in detergents to improve the shelf life and efficiency of the product but also as opacifiers to provide a “milky” texture to the product for improved consumer perception. While it can be argued that an improvement of the product efficiency and shelf life is necessary for its technical performance, the opacifiers’ function can be considered as “nice to have”, i.e., the product will perform just as well without opacifiers in the formulation. Therefore, when considering the suitability of alternatives, the evaluation should be focused on the necessary function(s) delivered by the substance of concern, and an alternative should not be disregarded if it does not provide those “nice to have” technical function(s).

As previously mentioned, it is not possible to evaluate whether uses of microplastics are necessary for the technical performance of the final product for seven uses, as not enough information about the service of the final products was provided in the restriction dossier, which made it difficult to understand the exact reasons why microplastics were used in the specific products. Furthermore, for two uses (i.e., for microplastics used in food additives and in paints and coatings), it is not possible to determine whether the functions provided by microplastics are necessary for the health and safety or critical for society because too many different final products were covered. For instance, in the restriction dossier, the use of “paints and coatings” covers not only products of microplastics for which it could be easily argued that a paint resistant to abrasion and to corrosion is necessary for health and safety (e.g., road markings, heavy duty industrial flooring) but also products which can easily be considered as not necessary for health and safety nor critical for the functioning of society (e.g., coating for furniture, decorative paints). These cases highlight the importance of providing concrete information linking the functions of a substance of concern with the services delivered by the specific final products in order to properly implement the “essential-use” concept. For example, if the dossier submitters do not have access to such information when preparing a restriction dossier, the industry should provide specific information, the latest during the public consultation, on the technical function of the substance of concern and how it relates to the end-uses and services, if they want to seek a derogation. Such an approach has already been taken for the ongoing restriction process of the uses of PFASs. The dossier submitters listed the uses of PFAS for which they did not manage to obtain adequate information to conclude on the needs for potential derogations and encouraged companies in the relevant sectors seeking for a derogation to provide information on their specific uses of PFAS during the public consultation.^45^

## 4 Implications and Outlook

This study analyzes how the information considered by the Competent Authorities in their decision-making of specific use exemptions/derogations/authorizations differs from the information which would be needed to implement the essential-use concept in the regulatory processes. The study suggests that implementing the essential-use concept requires no drastic changes in the information requirements, as most of the information necessary to perform an essential-use assessment is typically collected during the process. In addition, our analysis indicates that the essential-use concept could offer a valuable opportunity to inform decision-making on exemptions by shifting the focus toward evaluating the functions provided by a substance of concern and the services delivered by the end products, rather than solely assessing the risk associated with the specific uses. This is particularly relevant in cases where a risk-based approach may be inadequate, such as for persistent and/or bioaccumulative chemicals.

We acknowledge that the study did not evaluate how the essential-use concept could be implemented in a most effective and efficient manner. To operationalize the concept within legislation, further work is needed to address key implementation details, such as defining clear criteria of essentiality, determining to which groups of substances it should apply (e.g., end-use chemicals vs intermediates and feedstock substances), and identifying appropriate means to integrate the concept in the existing legislative framework.

Overall, this study contributes to the ongoing discussions of how the essential-use concept can support more transparent and goal-oriented chemical regulation to protect human health and the environment. By highlighting both the opportunities observed in current practices and that warrant further study, we hope this work can serve as a foundation for further efforts to refine and implement the concept in a way that ensures effective and efficient chemical management worldwide.

## Supplementary Material





## Notes

Extracted data supporting the analysis and conclusions of this paper, including the case study on intentionally added microplastics (PDF)
All the data collected to ensure the reproducibility of our work and our assessment and conclusions of the essentiality of these uses (XLSX)
