# Supplementary material for: Data Requirements for Implementing the “Essential-Use” Concept in Chemical Legislation
Source: Environ Sci Technol. 2025 Jun 2;59(22):10770–80. doi: 10.1021/acs.est.4c10866 (PMC12164256; doi:10.1021/acs.est.4c10866)
Supplement: Supplementary file 1 [file es4c10866_si_001.pdf]

Supplementary information to: Data Requirements for Implementing the "Essential-Use" Concept in Chemical Legislation

Romain Figuière[1*], Zhanyun Wang[2], Juliane Glüge[3], Martin Scheringer[3], Armin Siegrist[4,5], Ian T. Cousins[1]

[1] Department of Environmental Science, Stockholm University, SE-10691 Stockholm, Sweden

[2] Empa – Swiss Federal Laboratories for Materials Science and Technology and Society Laboratory, 9015 St. Gallen, Switzerland

[3] Institute of Biogeochemistry and Pollutant Dynamics, ETH Zürich, 8049 Zürich, Switzerland

[4] Institute of Environmental Engineering – Chair of Ecological Design, ETH Zürich, 8093 Zürich, Switzerland

[5] Institute of Food Nutrition and Health – Laboratory of Sustainable Food Processing, ETH Zürich, 8092 Zürich, Switzerland

*: Corresponding author: romain.figuiere@aces.su.se

Summary

Number of pages: 10

Number of Tables: 9 (4 are provided in the separate Excel files named *"Supporting data")*

Number of Figures: 1

Contents

S1. Data collected S2

S2. Essential-use concept criteria according to the guiding criteria of the essential-use concept from the European Commission[4] S2

S3. Background information the restriction dossier of intentionally added microplastics[5–7] S3

S4. Derogations as proposed by the Dossier submitter and evaluated by SEAC[5,8,9] S4

S5. Would the essential-use concept affect the proposed derogations? S6

References S10

S1. Data collected

See separate Microsoft Excel file.

Table S1.1 – Main reasons for granting exemptions under the Stockholm Convention (with quotes from POPRC)[1]

Table S1.2 – Main reasons for granting derogations under the REACH Restriction process (with quotes from SEAC)[2]

Table S1.3 – Overview of the applications for authorisation received under the REACH Authorisation process[3]

S2. Essential-use concept criteria according to the guiding criteria of the essential-use concept from the European Commission[4]

The tables S2.1 and S2.2 list the criteria that a use of a substance of concern should fulfil to be considered as “necessary for health and safety” and “critical for the functioning of society” (respectively) according to the latest essential-use concept framework proposed by WSP.

Table S2 1 Criteria for considering a use as "necessary for health and safety"[4]

Criteria	Additional information
**Preventing, monitoring or treating severe health issues**	Uses may include those in medical devices, pharmaceuticals, healthcare, or other health-related uses, directly linked to the prevention, monitoring, or treatment of severe health issues.<br>Mental illness should be included in "severe health issues" if patients affected by psychological problems have their abilities to engage in functional and occupational activities severely impaired
**Sustaining basic conditions for human life and health**	"Basic conditions for human health and life" include food, water, and shelter/security. Environmental health can be included here (e.g. if use to prevent air pollution)
**Managing and preventing health crises and emergencies**	E.g. Human health disease outbreak
**Personal safety**	Proper functioning of products/processes where the purpose of the chemical/product/process is to ensure personal safety (e.g. PPE, seatbelts, fire resistance products)
**Public safety**	Safety of public infrastructure (e.g. road safety, public building safety) as well uses required to ensure the effective functioning of emergency services to prevent danger to public safety (which could include, for example, military, police, anti-terrorism, cyber security, and fire safety services).
**Address a danger to animal health which cannot be contained by other means**	Safeguarding animal health and welfare in line with EU standards; Prevention and control of diseases (including zoonoses) and parasites; Prevention or minimisation of suffering caused to animals or pests, for example in the case of products used for pest control.

Table S2 2 Criteria for considering a use as "Critical for the functioning of society"[4]

Criteria	Additional info
**Providing resources or services which are critical for society**	I.e. resources (e.g. raw materials) and service which must remain in service for society to function (e.g. critical infrastructure in e.g. energy and transport; waste treatments; water treatments; communication infrastructure; healthcare infrastructure)
**Managing societal risks and impacts from natural and man-made crises and emergencies**	E.g. repairing/preventing damages to infrastructure in case of natural disaster
**Protecting cultural heritage**	"Cultural heritage" can be understood as (i) monuments, (ii) groups of buildings, (iii) sites.
**Running traditional and religious practices**	Applied in a similar way than in the Minamata Convention
**Protecting and restoring the natural environment**	E.g. To reduce emissions of greenhouse gases or biodiversity losses; for analysis and monitoring of pollutants; for remediation of pollutants in the environment

S3. Background information the restriction dossier of intentionally added microplastics[5–7]

In the restriction dossier, "microplastics" have been identified as *"small, usually microscopic, solid particles made of synthetic polymer"* which are associated with a long-term persistence in the environment as they are resistant to (bio)degradation. According to the dossier submitter, microplastics represent a concern for the environment and human health because: (i) they are small, which makes them available for ingestion and which increase their potential to be transferred along the food chain; (ii) they are very resistant to (bio)degradation, which result in microplastics being present in the environment for a long time after their release; (iii) they (bio)degrade progressively into smaller particles; (iv) they are very difficult to remove from the environment after release, which makes the microplastics pollution almost irreversible; and (v) (eco)toxicological hazard have been identified either from the polymers themselves or from the impurities of additives which are contained in them.

The dossier submitter identified that the main pathways for releases to the environment are direct releases to the environment, down-the-drain, or via municipal solid waste. To face this concern, the dosser submitter proposed this restriction in order to restrict the uses of "intentionally added" microplastics. Therefore, the microplastics which are formed in the environment via (bio)degradation of larger synthetic polymer-based articles (so-called "secondary microplastics") are not included in this restriction.

During the preparation of the restriction, the dossier submitter identified a wide diversity of technical functions of microplastics in various consumer, professional and industrial products in the following type of products and use sectors: Agriculture and horticulture (in fertilisers and plant protection products); Cosmetic (both rinse-off and leave-on cosmetic products); Detergents and maintenance

products; Paints, coatings and inks; Oil and gas; Construction; Medicinal products; Medical devices; and Food supplement and medical food.

In order to limit the socio-economic impacts of the restriction, the dossier submitter three different types of measures in the restriction: (i) a restriction on the placing on the market for those uses which inevitably result in releases to the environment, irrespective of the conditions of use; (ii) a labelling requirement for uses where releases of microplastics could occur if they are not used or disposed appropriately; and (iii) a reporting requirement to improve the quality of the information available.

As a result, the dossier submitter proposed that:

- the *"Polymers within the meaning of the Article 3(5) of Regulation (EC) No 1907/2006 shall not […] be placed on the market as a substance on its own or in a mixture as a microplastic in a concentration equal to or greater than [0.01] % w/w."*;
- From 18 months after the date of entry into force of the restriction, any manufacturer, importer or downstream user placing on the market a substance or a mixture containing microplastic for use derogated from the restriction shall "*ensure that the label and/or safety data sheet, where applicable, "instructions of use" and/or "package leaflet" provides […] any relevant instructions for use to avoid releases of microplastics to the environment*";
- From 12 months after the date of entry into force of the restriction, any downstream users using microplastics for uses derogated from the restriction shall "*send to ECHA […] by 31 January of each calendar year: (i) the identity of the polymer(s) used in the previous year; (ii) a description of the use of the microplastic; (iii) the quantity of microplastics used in the previous year; and (iv) the quantity of microplastics released in the environment, either estimated or measured in the previous year*".

S4. Derogations as proposed by the Dossier submitter and evaluated by SEAC[5,8,9]

Tables S1, S2, and S3 list the derogations which have been proposed by the dossier submitter, the main reason for such a proposal and the conclusion of SEAC. Within the restriction, the dossier submitter proposed six derogations from the whole restriction (Table S1); seven derogations from the ban only but for which the labelling requirements and mandatory reporting still apply (Table S2); and seven uses for which the dossier submitter proposed to set a date of entry into force later than the rest of the restriction (Table S3).

As illustrated in Figure 1, seven of these derogations were recommended either because no alternatives for the specific use were identified, or because the specific sector would not be able to fully implement the alternative before the initial date of entry into force, and it would need more time for the transition.

One derogation was proposed for the industrial uses of microplastics to make it clear that they are outside of the scope of this particular restriction. Three derogations were proposed for uses which are covered by other regulations (i.e. uses covered by regulations on fertilising products, on medicinal products for human or veterinary use, and on food additives). Two derogations were proposed for sludge and compost, and for food and feed as microplastics could be present as impurities, without being intentionally added to the mixture and/or product.

Six derogations were proposed for substances, mixtures and/or articles which do not contribute to the microplastic concern according to the dossier submitter, either because it does not cause harm to the environment due to its intrinsic properties (i.e. natural polymer which are not chemically modified, polymers which are (bio)degradable), and polymers with a water solubility above 2 g/L), or because it is assumed that the emissions to the environment are avoided (i.e. because microplastics are contained via technical means, or because the physical properties of the microplastics are permanently modified, or because microplastics are permanently incorporated into a soli matrix). At last, one derogation for the use on microplastics in *in vitro* diagnostic devices was proposed as the dossier submitter estimated that the releases of microplastics to the environment due to this use are very low, while the costs to substitute microplastics are rather high. Therefore, the dossier submitter assumed that the costs of implementing the restriction for this use would be too high compare to the potential benefits it would bring.

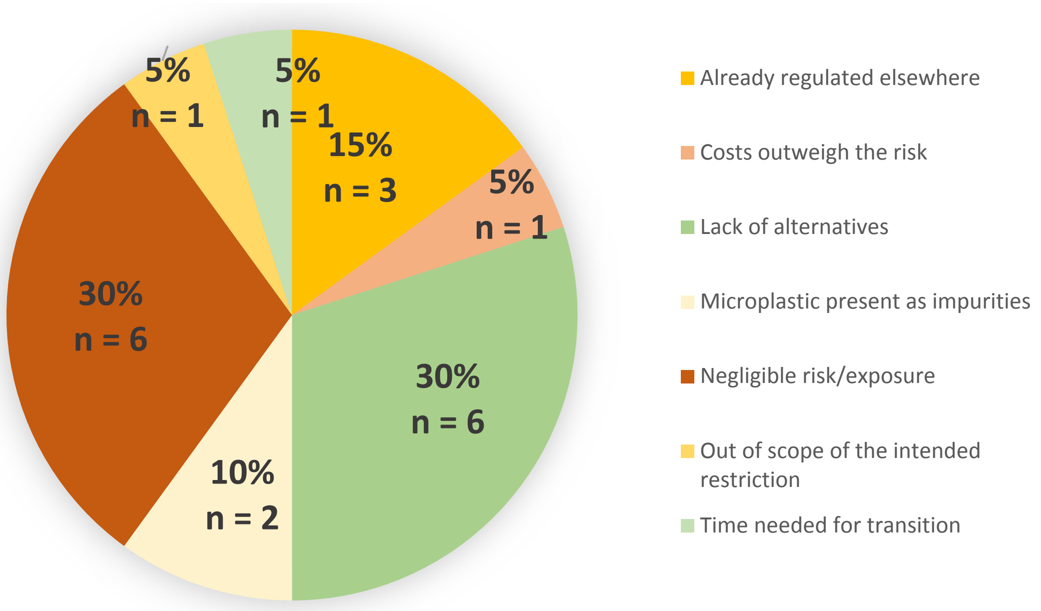

Figure S4 1 Main reasons for proposing a derogation to the restriction on intentionally added microplastics

S5. Would the essential-use concept affect the proposed derogations?

The Table S5 is provided in a separate Microsoft Excel file. It presents the detailed assessment of the essentiality of the uses of microplastics based on the information available in the restriction dossier.

Table S5 – Essentiality assessment of the uses of intentionally added microplastics

If the essential-use concept would be implemented in the REACH Restriction process in a similar way than in the Montreal Protocol, it can be assumed that a use which is necessary for health, safety and/or critical for the functioning of society and for which no safer alternatives are available could be derogated from a restriction. Following this assumption, one could wonder whether the same derogations would have been proposed by the dossier submitter of the microplastic restriction if they would have applied the essential-use concept. Table 1 lists all the derogations proposed by the dossier submitter in the microplastics restriction, the main reason for proposing such derogation, and whether such derogations would have been proposed if the essential-use concept would have been followed.

As previously explained, the dossier submitter proposed three derogations for uses and/or substances which are covered by other regulations in order to improve the implementation of the restriction and to avoid double regulations. It is likely that such type of derogations would still be proposed if the essential-use concept would be followed. Similarly, two derogations were proposed for those uses where microplastics are present as impurities in order to avoid unintended negative impacts of the restriction. It is also likely that the essential-use concept would not have changed the outcome of those derogations. One derogation was proposed by the dossier submitter to clarify that industrial uses of microplastics were not in the scope of the restriction. It is likely that such derogations would still be proposed if the essential-use concept would be implemented.

Three derogations have been proposed by the dossier submitter and by SEAC for those uses where the releases of microplastics are expected to be negligible either because microplastics are contained by technical means to prevent releases, because they are permanently contained in a solid matrix, or because their physico-chemical properties are permanently modified. If the essential-use concept would have been applied, it is unlikely that such derogation would have been proposed. Instead, the discussion would have been focused on the essentiality of such uses. Similarly, in the particular case of the derogation for use of microplastics in IVD kits, the fact that releases of microplastics from this use are relatively small would not have weighed in the balance in the decision-making. Instead, the assessment would have focused on the essentiality of the technical function of microplastics for the IVD kits to determine whether a derogation should be proposed or not.

At last, the dossier submitter proposed seven derogations for uses for which alternatives would not be sufficiently available to replace microplastics by the date of entry into force of the restriction. Therefore, it can be assumed that those uses would have met one of the two conditions to be considered essential. If the essential-use concept would have been implemented at the time of the restriction, it is likely that

the dossier submitter would have evaluated whether each of those uses are necessary for health, safety and/or critical for the functioning of society before proposing a derogation, which could have changed the outcome of the decision on the potential derogation.

Table S5 1 Derogations from the whole restriction as proposed by the dossier submitter, and with SEAC conclusion

**Derogation as proposed by the dossier submitter**	**Main reason for derogation**	**Quotes (from final opinion)**	**SEAC conclusion**
Natural polymers not chemically modified	Negligible risk/exposure	"natural polymers, as long as their chemical structure has not been chemically modified, are exempt from the restriction as they are inherently biodegradable and therefore do not contribute to the microplastics concern." p.82	Agreed
Polymers which are (bio)degradable	Negligible risk/exposure	"(bio)degradable polymers are exempt from the restriction on the basis that they do not contribute to the microplastic concern, even though they could remain in the environment for some time after use/release." p.82	Agreed
Polymers with solubility > 2g/L	Negligible risk/exposure	"including an additional derogation for water soluble polymers would improve the targeting of the restriction since soluble polymers do not contribute to the identified risk" p.100	Not assessed by SEAC, but RAC agreed
Substances or mixtures regulated under Regulation (EC) No. 2019/1009 on Fertilising Products	Already regulated elsewhere	"Complete derogation of EU regulated fertilisers from the scope of the restriction to avoid double regulation." p.82	Agreed
Sludge and compost	Microplastic present as impurities	"Complete derogation from the scope of the restriction as this was not intended to be part of the scope. Microplastics are not intentionally added into sludge and composts." p.82	Agreed
Food and feed	Microplastic present as impurities	"As these can unintentionally contain microplastics above the specific concentration limit then it is prudent to ensure that they are specifically derogated." p.82	Agreed

Table S5 2 Derogations from the ban only as proposed by the dossier submitter, and with SEAC conclusion

Derogation as proposed by the dossier submitter	Main reason for derogation	Quotes (from final opinion)	SEAC conclusion
Substances or mixtures containing microplastics used at industrial sites	Out of scope of the intended restriction	"This is required to allow continued use at industrial uses" p.82	Agreed
Medicinal products for human or veterinary use	Already regulated elsewhere	"Derogation from the scope of the restriction on use to avoid potential double regulation and any risk that the availability of medicines could be affected." p.82	Agreed
Substances or mixtures containing food additives	Already regulated elsewhere	"Derogation from the scope of the restriction on use to avoid potential double regulation, and market-distortion." p.83	Agreed
In-vitro diagnostic devices	Costs outweigh the risk	"Derogation from the scope of the restriction on use based on cost-effectiveness and socio-economic considerations." p.83	Agreed
Substances, mixtures or articles where microplastic is contained by technical means to prevent releases	Negligible risk/exposure	"Generic derogation from the restriction for uses where OC and RMM are implemented that are appropriate to adequately control the risk from the use of microplastics." p.83	Agreed
Substances, mixtures or articles where the physical properties of the microplastic are permanently modified	Negligible risk/exposure	"Generic derogation from the restriction for uses of microplastics as a substance or in a mixture where the microplastics are 'consumed' or otherwise permanently cease to exist at the point of end use" p.83	Agreed
Substances, mixtures or articles where the microplastics are permanently incorporated in a solid matrix	Negligible risk/exposure	"Generic derogation from the restriction for uses of microplastics as substances or mixtures where the microplastics are permanently 'contained' at the point of use. Permanence is intended to relate to the useful (service) life of the solid matrix, not the waste life-cycle stage." p.84	Agreed

Table S5 3 Derogations for a longer transitional period as proposed by the dossier submitter, and with SEAC conclusion

Derogation as proposed by the dossier submitter	Main reason for derogation	Quotes (from final opinion)	SEAC conclusion
Medical devices (where microplastics cannot be contained during end use)	Lack of alternatives	"a transition period of 6 years is considered to allow for sufficient time to reformulate and transition to alternatives." p.104	Agreed
Rinse-off cosmetic products	Time needed for transition	"Reformulations are the most important factor in this case. The typical reformulation process takes 2.5-4.5 years. Alternatives are widely available." p.105	Agreed
Detergents and maintenance products without microbeads	Lack of alternatives	"Reformulations are the most important factor in this case." p.105	Agreed
Fragrance encapsulates	Lack of alternatives	"During the Annex XV consultation industry provided information on the substitution process of microplastics in fragrance encapsulation systems, which the Dossier Submitter found may justify a longer transition period of 8 years for this use" p.105	Agreed
Agridultural & horticultural uses: Controlled release fertilisers & fertiliser additives	Lack of alternatives	"Time is required for the development of biodegradable polymers. The transitional period is intended to align with the new Fertilising Products Regulation" p.106	Agreed
Agridultural & horticultural uses: Capsule suspensions PPPs & coated seeds	Lack of alternatives	"Time is required for the development of biodegradable polymers, whose functionalities might be different from products covered under the FPR"	Agreed
Leave-on cosmetic products	Time needed for transition	"Reformulations are the most important factor in this case." p.107	Agreed

References

(1) United Nations Environment Programme. *The New POPs under the Stockholm Convention*. Stockholm Convention website. https://chm.pops.int/?tabid=2511 (accessed 2023-11-16).

(2) European Chemicals Agency. *Registry of restriction intentions*. ECHA website. https://echa.europa.eu/registry-of-restriction-intentions (accessed 2023-11-16).

(3) European Chemicals Agency. *Main alternatives to harmful substances subject to REACH authorisation*. ECHA website. https://echa.europa.eu/alternatives-to-harmful-substances-subject-to-authorisation (accessed 2023-12-13).

(4) European Commission. *Communication from the Commission – Guiding criteria and principles for the essential use concept in EU legislation dealing with chemicals*. https://eur-lex.europa.eu/eli/C/2024/2894/oj (accessed 2024-06-17).

(5) European Chemicals Agency. *Annex XV Restriction Report on Intentionally Added Microplastics*; 2019. https://echa.europa.eu/documents/10162/05bd96e3-b969-0a7c-c6d0-441182893720 (accessed 2024-10-04).

(6) European Chemicals Agency. *Annex to Annex XV Restriction Report on Intentionally Added Microplastics*; 2019. https://echa.europa.eu/documents/10162/db081bde-ea3e-ab53-3135-8aaffe66d0cb (accessed 2024-10-04).

(7) European Chemicals Agency. *Registry of restriction intentions until outcome - Microplastics*. https://echa.europa.eu/registry-of-restriction-intentions/-/dislist/details/0b0236e18244cd73 (accessed 2024-02-21).

(8) European Chemicals Agency. Final Background Document of the Restriction Dossier on Intentionally Added Microplastics. **2020**.

(9) European Chemicals Agency. *Committee for Risk Assessment and Committee for Socio-Economic Analysis Opinion on an Annex XV Dossier Proposing Restrictions on Intentionally-Added Microplastics*; 2020. https://echa.europa.eu/documents/10162/a513b793-dd84-d83a-9c06-e7a11580f366 (accessed 2024-10-04).
